# Pyrethroid as a Substance of Abuse

**DOI:** 10.1155/2014/169294

**Published:** 2014-11-06

**Authors:** Pravesh Sharma, Stephen Manning, Regina Baronia, Saira Mushtaq

**Affiliations:** Texas Tech University Health Sciences Center, 3601 4th Street, Stop 8103, Lubbock, TX 79430, USA

## Abstract

This is a case of a 22-year-old Hispanic male with a history of bipolar disorder and methamphetamine dependence who was admitted after presenting with suicidal ideations by slashing his throat with a machete. The patient had been smoking and inhaling “processed” pyrethroid for about eight weeks as an inexpensive methamphetamine substitute. He reported experiencing a “rush” similar to methamphetamine after using pyrethroid from liquid insecticide that had been heated (electrocuted) or sprayed on hot metal sheets until it crystallized. The patient presented with no significant physical markings or findings but claimed to have his suicidal ideations precipitated by concerns of ill effects of pyrethroid on his health. He also had positive urine drug screen for methamphetamine, which he admitted to using on the day of admission. We conclude that it is important for physicians to maintain a high level of suspicion for alternate and uncommon substances of abuse as well as risks for suicidal tendencies in these patients.

## 1. Introduction

Pyrethrum has been used for many years as a botanical insecticide. The term “pyrethrum” refers to the dried and powdered flower heads of a white-flowered, daisy-like plant belonging to the* Chrysanthemum* genus. Pyrethrins are prepared from dried* Chrysanthemum cinerariaefolium* and/or* Chrysanthemum cineum* flower heads. There are certain limitations of pyrethrum extract, also known as pyrethrins, such as high rate of photodegradation and a short “knockdown” (rapid paralysis) effect. This led to the discovery of synthetic derivatives of pyrethrins which are more resistant to photodegradation as compared to their parent compounds.

## 2. Case Report

Mr. A, a 22-year-old Hispanic male with past psychiatric history of methamphetamine dependence and bipolar disorder type 1, came to the emergency department because of suicidal ideation with a plan to slash his throat with a machete. The patient was very agitated in triage and during initial assessment. In the emergency room (ER), the patient reported that he had been using methamphetamine for the last two years. His last use was a few hours before coming to the ER and that was his only use during the past six to eight weeks.

On physical examination, temperature was found to be 96.5 degree Fahrenheit, pulse 105/minute, blood pressure 124/78 mm Hg, weight 68.0 kg, and height 173 cm. His pupils were 5 mm in diameter and reactive. In the review of systems, the patient denied any pulmonary, cardiac, renal, and abdominal complaints. There was no increased lacrimation. Lungs were clear and the heart rate was regular without murmurs. Bowel sounds were normoactive. The patient was not oriented to time. His affect was flat and irritable. He was evasive and tangential while answering questions. A chest radiograph and electrocardiogram were normal. His urinary drug screen was positive for amphetamines, methamphetamines, and cannabinoids. Blood chemistry concentrations/counts/percent of the following analyses were mainly within reference limits: alcohol <5 mg/dL, salicylate <4 mg/dL, sodium 141 mmol/L, potassium 3.8 mmol/L, chloride 107 mmol/L, blood urea nitrogen 10 mg/dL, creatinine 0.9 mg/dL, aspartate transaminase 23 U/L, alanine transaminase 18 U/L, alkaline phosphatase 69 U/L, thyroid stimulating hormone 0.57 *μ*IU/mL, white blood cells 10.7 K/uL, red blood cells 4.49 Mu/L, haemoglobin 13.9 g/dL, hematocrit 42.2%, and platelets 239 K/uL. Urinalysis revealed nothing abnormal.

The patient was transferred to the inpatient psychiatry unit. The next day, the patient was disoriented, refused to talk to the treatment team, and slept most of the day. On his third hospital day, the patient was much more oriented and reported that he was unable to get methamphetamine for the past six to eight weeks because he could not afford it. He claimed to use an insecticide, mostly cockroach killer, to “get high.” He would either “electrocute” the bottle or spray it on a heated metal sheet, and when the contents of the bottle turned into crystal, he would either inhale it or smoke it. The patient would use the crystals formed from one bottle for four to seven days. He claimed that his friends used those crystals intravenously after diluting them. The patient reported that it would give him the same high as methamphetamine and he would occasionally have feelings of deja vu. The patient also reported having olfactory hallucinations when he was on “crystal roach killer” but could not elaborate on that. He said that he liked the increase in heart rate and the “rush” he used to get after every use.

The patient's last use of the “crystal roach killer” was three days before coming to the ER. The patient reported that he would sleep most of the day when he was using it. He denied unwanted adverse effects during his use except for frequent headaches upon waking up which were relieved by over-the-counter analgesics. The patient had been using the insecticide for six to eight weeks and only used methamphetamine one (1) day before coming to the ER. The patient claimed that he had been compliant with his medications for bipolar disorder until he started using “crystal roach killer.” The patient was taking divalproex sodium 500 mg twice daily and quetiapine extended-release 300 mg at bedtime. He voiced that he, since a few weeks before coming to the ER, got scared that he might get cancer because of the frequent use of the insecticide, which led him to have suicidal ideation.

## 3. Discussion

Pyrethroids are known to cause hyperexcitation by targeting sodium channels which are kept open for unusually long periods of time [1]. Animal studies of pyrethroid toxicity have shown hyperglycemia and elevated plasma levels of noradrenaline and adrenaline [2]. These may account for the “rush” the patient experienced with his “crystal roach killer.” Ingestion and parenteral injection of pyrethroids in suicide attempts, occupational exposure, and accidents are well documented and have resulted in poisoning syndromes with characteristic sympathetic activation, lacrimation, hyperexcitability, choreoathetosis, and status epilepticus [3–5].

Reports of association of pyrethroids with parenteral drug abuse are fairly sparse. These have shown adverse effects like local erythema, cellulitis, and vasculitis [6–9]. These reported cases had the pyrethroids injected either subcutaneously (popping) or intravenously usually resulting in local effects that could be noted immediately on examination. There was also association with suicidal history or ideation. Communication via phone with the National Pesticide Information Center (NPIC) was made. NPIC stated that they had no official documented cases processing pyrethroid to produce effects similar to methamphetamine or case reports of the use of pyrethroid as a recreational substance.

There are certain limitations to this report. The anamnesis was taken from a person suffering from substance abuse and bipolar disorder, the latter being untreated for the last six to eight weeks, making his credibility questionable. However, our longstanding knowledge of patients with this kind of problems in this area suggests that this patient's report should not be dismissed without careful consideration. The suicidal thoughts emerging in the patient may also not be a result of the use of pyrethroid only, as the patient's untreated bipolar disorder may have made him more prone to such ideation.

This case report is interesting in the fact that the pyrethroid was “processed” so that it could be smoked or inhaled to get a feeling of “rush.” Despite the reported use for six to eight weeks, the patient had no significant physical findings associated with pyrethroid abuse when he presented to the ER. The patient had suicidal ideations apparently precipitated by his use of “crystal roach killer.” His statements about friends using it intravenously highlight the phenomenon of an underreported substance being abused with little or no telltale signs by people with high risk for self-harm. It is important for physicians to maintain a high level of suspicion for alternate and uncommon substances of abuse and suicidal ideation among people who abuse these substances.
